# Supplementary material for: PhyBin: binning trees by topology
Source: PeerJ. 2013 Oct 22;1:e187. doi: 10.7717/peerj.187 (PMC3807594; doi:10.7717/peerj.187)
Supplement: Table S1 — The number of clusters and cluster membership when PhyBin is run on the Wolbachia ortholog set (503 trees total, –editdist=3) using single linkage (–single), UPGMA (–UPGMA [default]), or complete linkage (–complete) hierarchical clustering. [file peerj-01-187-s001.docx]

**Supplementary Table 1.** The number of clusters and cluster membership when PhyBin is run on the *Wolbachia* ortholog set (503 trees total, --editdist=3) using single linkage (--single), UPGMA (--UPGMA [default]), or complete linkage (--complete) hierarchical clustering.

| Hierarchical Clustering Method | Cluster Membership |
| --- | --- |
| Single linkage (nearest neighbor) | 456,5,2,2,2,1,1,1,1,1,1,1,1,1,1,1,1,1,1,1,1,1,1,1,1,1,1,1,1,1,1,1,1,1,1,1,1,1,1,1,1 |
| UPGMA (average neighbor) | 34,29,27,21,20,19,19,17,15,13,13,13,13,13,11,11,8,8,8,8,7,6,5,5,5,5,4,4,4,4,4,4,4,3,3,3,3,3,3,3,3,3,3,3,2,2,2,2,2,2,2,2,2,2,2,2,2,2,2,2,2,2,2,2,1,1,1,1,1,1,1,1,1,1,1,1,1,1,1,1,1,1,1,1,1,1,1,1,1,1,1,1,1,1,1,1,1,1,1,1,1,1,1,1,1,1,1,1,1,1, |
| Complete linkage (furthest neighbor) | 34,27,20,19,19,17,15,13,13,12,11,11,11,10,10,9,8,8,8,8,7,6,6,6,5,5,5,4,4,4,4,4,3,3,3,3,3,3,3,3,3,3,3,2,2,2,2,2,2,2,2,2,2,2,2,2,2,2,2,2,2,2,2,2,2,2,2,2,2,2,2,2,2,1,1,1,1,1,1,1,1,1,1,1,1,1,1,1,1,1,1,1,1,1,1,1,1,1,1,1,1,1,1,1,1,1,1,1,1,1,1,1,1,1,1,1,1,1,1,1,1,1,1,1,1,1,1,1,1,1,1,1,1,1,1,1,1,1,1,1 |
